# Curcumin Sensitizes Colorectal Cancer Cells to TRAIL-Mediated Apoptosis

**DOI:** 10.3390/ijms27219444

**Published:** 2026-10-23

**Authors:** Erika Rimondi, Marta Manfredini, Francesca Nigro, Arianna Gonelli, Daniela Milani, Claudio Celeghini, Maurizio Previati, Annalisa Marcuzzi

**Affiliations:** 1 Department of Translational Medicine and LTTA Centre, University of Ferrara, 44121 Ferrara, Italy; 2 Department of Translational Medicine, University of Ferrara, 44121 Ferrara, Italy; marta.manfredini@unife.it (M.M.); francesca.nigro@unife.it (F.N.); daniela.milani@unife.it (D.M.); claudio.celeghini@unife.it (C.C.); maurizio.previati@unife.it (M.P.); annalisa.marcuzzi@unife.it (A.M.); 3 Department of Environmental Sciences and Prevention, University of Ferrara, 44121 Ferrara, Italy; arianna.gonelli@unife.it

**Keywords:** colon cancer, TRAIL, curcumin, pyroptosis

## Abstract

Colorectal cancer (CRC) remains a major clinical challenge due to resistance to conventional cell death-inducing therapies. TRAIL selectively induces apoptosis in cancer cells via death receptors, but its effectiveness in preclinical models is limited by tumor resistance mechanisms. Curcumin, a natural polyphenol, modulates multiple survival and apoptotic pathways and has been proposed as a sensitizing agent to overcome TRAIL resistance. Here, we investigated the combinatorial effects of TRAIL and curcumin in the HCT116 colorectal cancer cell model. Cells were treated with TRAIL, curcumin, or their combination for 24 and 48 h, evaluating apoptosis, viability, and underlying mechanisms. Curcumin significantly enhanced TRAIL-induced apoptosis (*p* < 0.0001) and further reduced MTT-derived metabolic activity (*p* < 0.01) compared with TRAIL treatment alone. This effect remained evident after 48 h, although the stability and persistence of TRAIL activity were not directly assessed. Mechanistically, combined treatment enhanced PARP cleavage, γ-H2AX phosphorylation, and BAX and Caspase-3 cleavage, while downregulating anti-apoptotic IAP family members. Notably, cotreatment also promoted pyroptosis, shown by characteristic morphological changes (*p* < 0.0001) and increased Gasdermin-E cleavage. These findings indicate that curcumin sensitizes CRC cells to TRAIL by engaging both apoptotic and pyroptotic pathways, suggesting a potential approach to counteract both TRAIL and multidrug resistance in colorectal cancer.

## 1. Introduction

Tumor Necrosis Factor-Related Apoptosis-Inducing Ligand (TRAIL), a member of the TNF superfamily, has long been proposed as an anti-cancer treatment for its ability to induce apoptosis specifically in cancer cells by binding to death receptors DR4 and DR5 [1,2,3], triggering the extrinsic apoptotic pathway, without harming normal cells [4,5]. Several clinical trials have confirmed the safety and low toxicity of TRAIL, but also its limited anti-tumor efficacy [6,7,8] due to its short half-life or due to the intrinsic or acquired resistance of tumor cells to TRAIL. This includes the reduction in death receptors DR4/DR5, the concomitant increase in decoy receptors DcR1/DcR2 and the upregulation of c-FLIP and IAP family members [9,10]. Consequently, combination strategies have been explored to enhance TRAIL efficacy, including its co-administration with curcumin [11].

Curcumin, a polyphenol derived from turmeric (*Curcuma longa*), has been reported to exert multiple biological effects in cellular homeostasis. In cancer cells, it acts as a multi-target agent, interfering with key features of tumorigenesis such as proliferation, evasion of apoptosis, angiogenesis, invasion, metastasis, and chemoresistance [12,13,14,15]. These broad-spectrum effects, observed across different cancer cell lines (e.g., gastrointestinal, breast, lung), are mediated through the modulation of critical signaling pathways. Curcumin both suppresses pro-survival pathways (e.g., NF-κB, PI3K/Akt, STAT3) and activates pro-apoptotic and tumor suppressor pathways (e.g., p53, caspase cascade) [14]. In contrast, in healthy cells, curcumin has been reported not to exert these cytotoxic effects and to promote cellular homeostasis and tissue repair, and to reduce lipid peroxidation, attenuating oxidative stress [15].

While curcumin has shown a lot of promise in early lab studies, translating these results into clinical success has been tough. The main challenge is its poor bioavailability, which limits absorption and biological effect [16]. Combination strategies with existing drugs have been proposed as a way to achieve biological effects at lower curcumin concentrations; however, they do not directly resolve the pharmacokinetic limitations of curcumin, which are better addressed by dedicated delivery systems.

However, curcumin is a controversial compound. Owing to its chemical instability, reactivity, and tendency to aggregate and interfere with several assay readouts, it has been classified as a pan-assay interference compound (PAINS) and its conclusive clinical efficacy is the subject of many studies to evaluate its therapeutic prospects [17].

Thus, the combination of TRAIL, which activates the extrinsic apoptotic pathway through interaction with death receptors DR4/DR5 [1], and curcumin, which modulates different intracellular survival pathways, including NF-κB, c-FLIP and IAPs [18], may potentiate apoptotic response in colorectal cancer cells by concurrently activating death receptor signaling and inhibiting anti-apoptotic mechanisms [11,19].

Colorectal cancer (CRC) remains a massive global health challenge, largely due to the persistent issue of drug resistance: therefore, finding new solutions is critical. Despite significant progress in surgical and pharmacological interventions, treatment failure in CRC remains a major clinical challenge, due to the development of acquired resistance to conventional therapies [20]. Tumor cells often evade apoptosis through the upregulation of pro-survival and anti-apoptotic pathways, limiting therapeutic efficacy. For this reason, identifying novel strategies able to overcome drug resistance represents a key priority in CRC research [21].

Previous studies have demonstrated the ability of curcumin to sensitize cancer cells to TRAIL treatment, inducing the upregulation of TRAIL receptors [11,22]. In particular, it has been proven that DR5 upregulation induced by curcumin is mediated by reactive oxygen species (ROS) activation. Based on this evidence, the present research evaluates whether the combined anti-tumoral properties of TRAIL and curcumin could enhance the effect of the single treatment and promote cancer cell death, focusing on the treatment-specific signaling pathways.

In this context, we investigated the effects of these molecules in an in vitro model, using the HCT116 cell line, a reliable and well-documented model for studying how cancer cells evade death and resist to treatment, thereby demonstrating for the first time that cotreatment, in addition to inducing apoptotic signaling pathways, also promoted pyroptosis.

## 2. Results

### 2.1. Curcumin Potentiates the Pro-Apoptotic Activity of TRAIL

**Figure 1:**
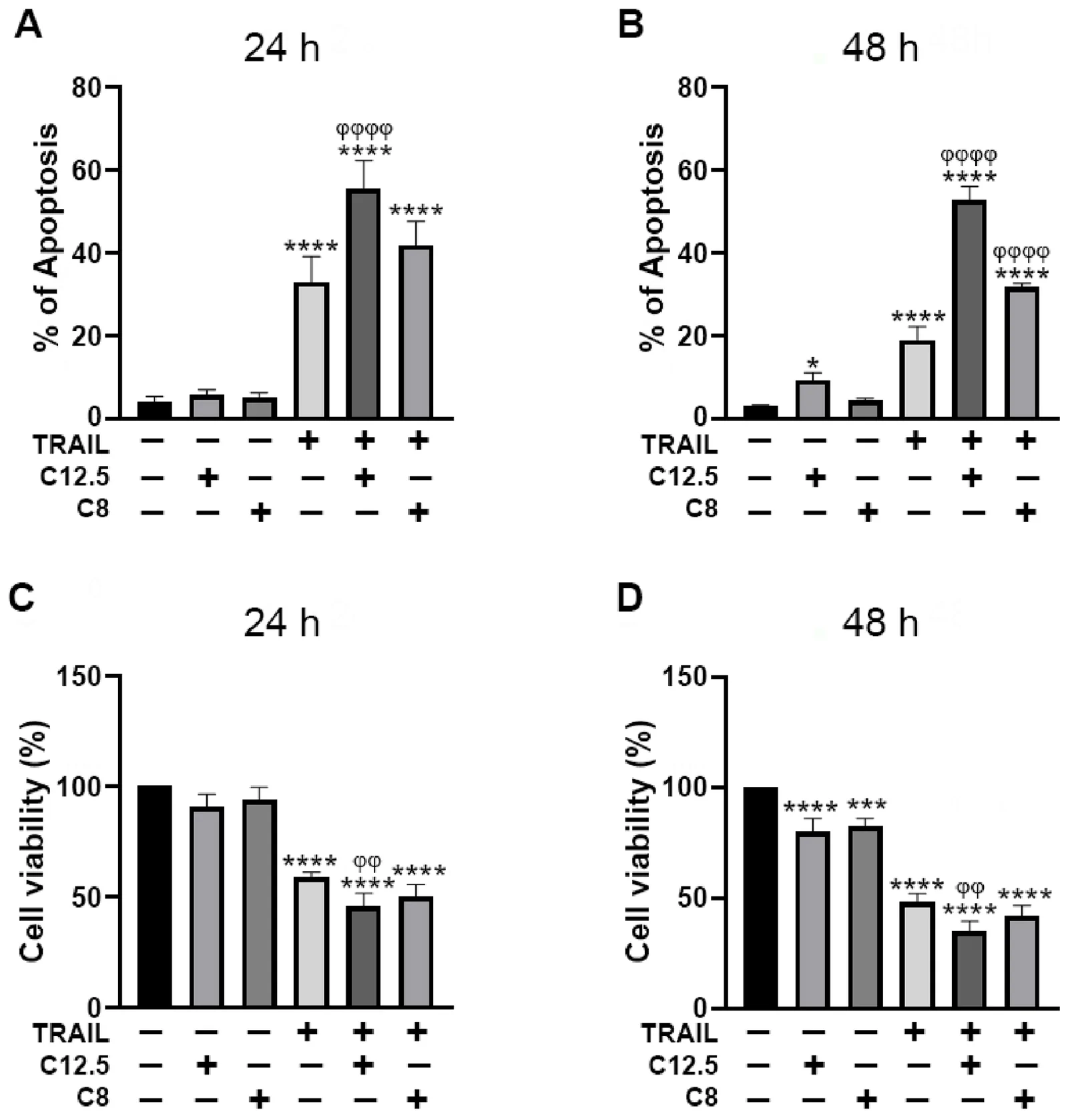
Apoptosis and cell viability evaluation in HCT116 cells treated with TRAIL alone or in combination with two different concentrations of curcumin. The graphs show the apoptosis (**A**,**B**) and the cell viability values (**C**,**D**) obtained by treating HCT116 cells with TRAIL 500 ng/mL, 12.5 µM and 8 µM curcumin, and their combinations, compared to untreated cells (set to 100%), after 24 and 48 h of incubation. (**A**,**B**) Results of four (**A**) and three (**B**) different experiments are reported as mean ± SD. (**C**,**D**) Results of four different experiments are reported as mean ± SD. Statistical significance vs. untreated: * *p* < 0.05; *** *p* < 0.001; **** *p* < 0.0001. Statistical significance vs. TRAIL: ^φφ^ *p* < 0.01; ^φφφφ^ *p* < 0.0001.

In a first set of experiments, we evaluated the ability of TRAIL and curcumin, used alone or in combination, to induce apoptosis in HCT116 cells. After 24 and 48 h of treatment the cells were harvested and analyzed in flow cytometry by using the Annexin V/PI assay. As shown in Figure 1A, after 24 h of treatment, curcumin alone, at both concentrations, did not increase the number of apoptotic cells compared to the untreated control. As expected, treatment with TRAIL 500 ng/mL significantly induced apoptosis in HCT116 cells, leading to a significant increase in apoptosis percentage compared to untreated cells. A significant induction of apoptosis was also observed with the combined treatment with TRAIL and 12.5 µM or 8 µM curcumin. Of note, the combination of TRAIL and 12.5 µM curcumin was able to increase the pro-apoptotic effect observed with TRAIL alone, suggesting that this concentration of curcumin can potentiate the apoptotic response elicited by TRAIL.

After 48 h from administration (Figure 1B), we observed that 12.5 µM curcumin alone, but not 8 µM, was able to induce a slight but significant increase in the percentage of apoptotic cells compared to the untreated condition. On the other hand, TRAIL pro-apoptotic activity was drastically reduced, although the percentage of apoptotic cells was still significantly higher compared to the control, but not to 12.5 µM curcumin. Of note, contrary to TRAIL, TRAIL + 12.5 µM curcumin did not suffer from the same loss of activity and exerted a strong pro-apoptotic effect, even comparable to that observed at 24 h, suggesting that the combination of the two molecules was able to compensate the reduced activity of TRAIL. Instead, TRAIL + 8 µM curcumin seems to be moderately affected, as it elicited a stronger apoptotic response than TRAIL alone, even if not comparable to TRAIL + 12.5 µM curcumin.

The analysis of apoptosis was accompanied by a reduction in MTT-derived metabolic activity. As shown in Figure 1C,D, the treatment with TRAIL alone or in combination with both curcumin concentrations (12.5 µM and 8 µM) significantly reduced the cell viability compared to untreated cells at 24 h and at 48 h after treatments. As expected, the combination of TRAIL and 12.5 µM curcumin was able to significantly lower the viability also with respect to TRAIL at 24 h and at 48 h after treatments.

### 2.2. TRAIL-Based Treatments Increase Expression of Apoptotic Markers

The pro-apoptotic effect of the combinatorial treatment TRAIL + curcumin was also evaluated by analyzing two well-established markers of apoptosis and genotoxic stress, PARP and γ-H2AX.

**Figure 2:**
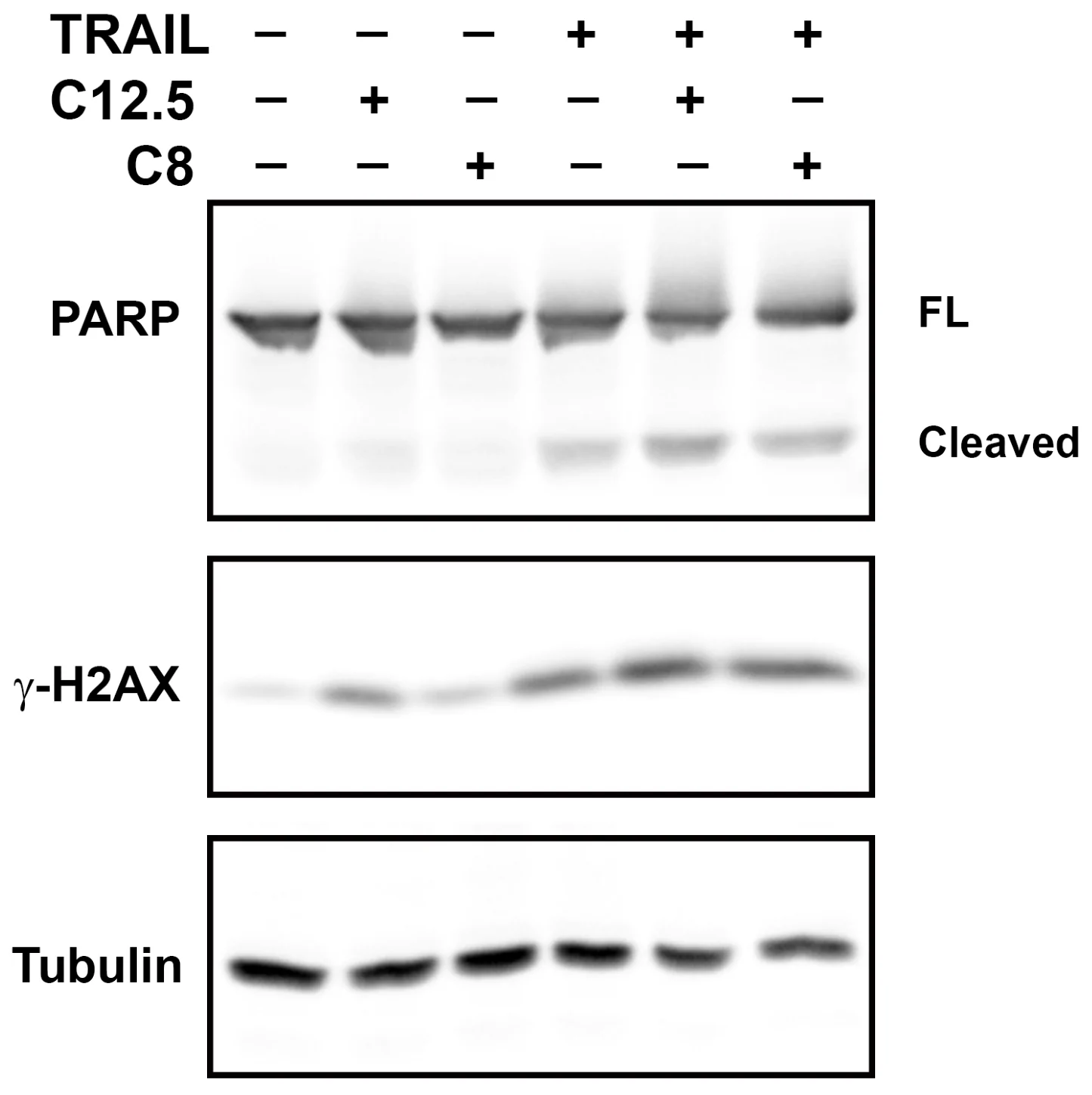
Western Blot analysis of PARP cleavage and γ-H2AX expression in HCT116 after 24 h of treatment. Representative images of Western Blot results for PARP and γ-H2AX, performed on HCT116 cells following treatment with TRAIL, 12.5 µM and 8 µM curcumin, and their combinations for 24 h. Tubulin was used as loading control.

As shown in Figure 2, TRAIL was able to induce the cleavage of PARP, with the reduction in the full-length form and increase in the cleaved fragment, and this effect was greater when TRAIL was combined with 12.5 µM curcumin (Figure 2).

Another apoptotic marker analyzed was γ-H2AX, the phosphorylated form at Serine 139 of the histone protein H2AX. As previously observed for PARP cleavage, H2AX phosphorylation was also more induced when cells were co-treated with TRAIL and 12.5 µM curcumin (Figure 2), consistent with the possibility that cotreatment improves its pro-apoptotic effect on CRC cells.

Of note, curcumin alone was able to induce a slight increase in γ-H2AX only when used at a concentration of 12.5 µM.

Based on these preliminary results we selected 12.5 µM as the concentration of use for curcumin in the next experiments.

### 2.3. Signaling Pathways Mediating Apoptosis

**Figure 3:**
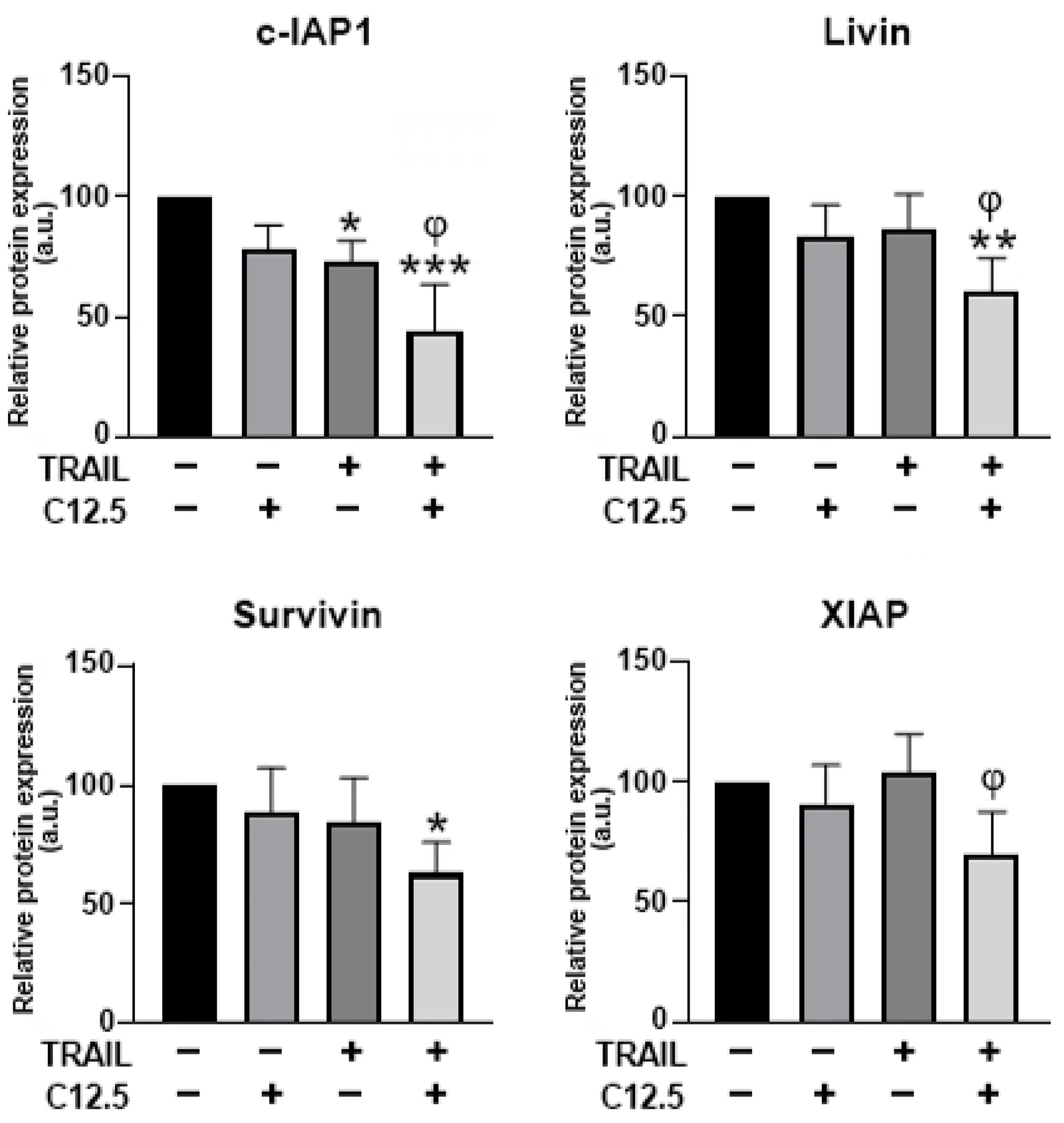
Evaluation of multiple apoptosis-related proteins in HCT116 after 24 h of treatment. HCT116 cells were treated for 24 h with TRAIL alone or in combination with 12.5 µM curcumin. Results of four independent experiments are presented as % with respect to untreated set to 100% and are reported as mean ± SD. Statistical significance vs. untreated: * *p* < 0.05; ** *p* < 0.01; *** *p* < 0.001. Statistical significance vs. TRAIL: ^φ^ *p* < 0.05. Relative protein expression is expressed in arbitrary units (a.u.).

In order to define the molecular mechanisms activated by TRAIL, curcumin, and their combination to elicit apoptosis, we analyzed the expression of proteins involved in apoptotic pathways, after 24 h of treatment.

c-IAP1 showed a significant reduction in protein levels after treatment with TRAIL and its combination with 12.5 µM curcumin with respect to untreated cells. Moreover, the combined treatment also induced a significant decrease compared to TRAIL alone (Figure 3).

The anti-apoptotic proteins Livin, Survivin and XIAP showed a similar modulation, with a significant reduction in protein levels observed only when cells were treated with TRAIL + curcumin (Figure 3).

**Figure 4:**
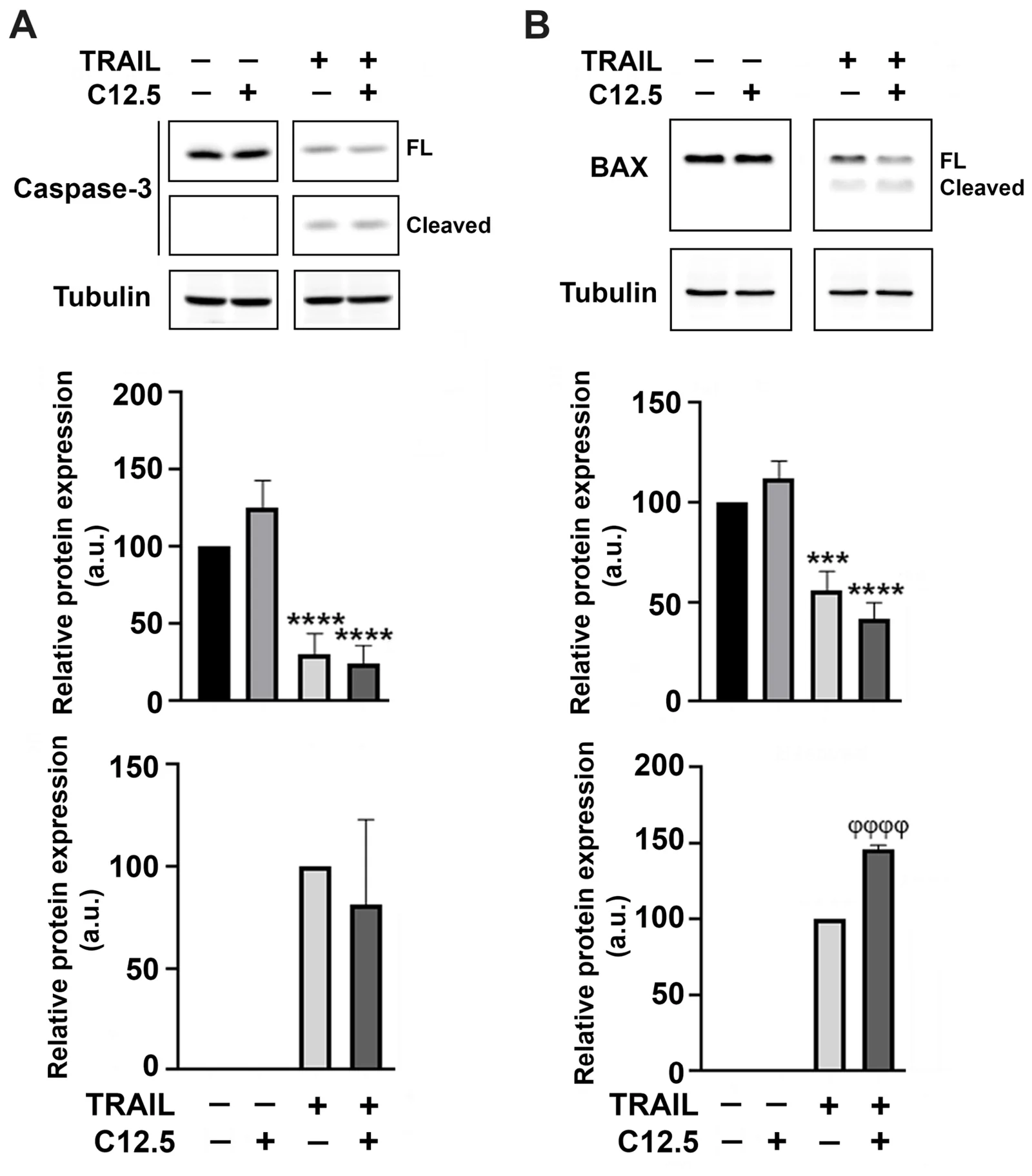
Western Blot analysis of Caspase-3 and BAX cleavage in HCT116 after 24 h of treatment. Representative images of Western Blot results for Caspase-3 (**A**) and BAX (**B**), performed on HCT116 cells following treatment with TRAIL alone or in combination with 12.5 µM curcumin for 24 h. Tubulin was used as loading control. FL = full-length protein. The band densitometry was quantified using the iBright Analysis Software and expressed in relative protein expression indicated in arbitrary units (a.u.). Full-length proteins were normalized to the untreated band set to 100%. Cleaved proteins were normalized to the TRAIL band set to 100%. Statistical significance vs. untreated: *** *p* < 0.001; **** *p* < 0.0001. Statistical significance vs. TRAIL: ^φφφφ^ *p* < 0.0001.

The cleavage of Caspase-3, the effector caspase activated by both extrinsic and intrinsic apoptotic pathways, was evaluated with Western Blotting. As shown in Figure 4A, curcumin alone was not sufficient to activate Caspase-3; conversely, TRAIL, alone or in combination with curcumin, induced the cleavage of full-length Caspase-3 (35 kDa) into its active form (17 kDa). We also observed a decrease in p21 BAX levels (FL), which corresponded to an increase in the cleaved form of this protein (p18), when HCT116 cells were treated with TRAIL alone or in combination. Notably, the proteolytic cleavage of BAX was significantly increased in HCT116 cells treated with TRAIL in combination with 12.5 µM curcumin with respect to cells treated with TRAIL alone (Figure 4B).

Furthermore, treatments with TRAIL, alone or in combination with curcumin, also modulated anti-apoptotic signals (Figure S1).

### 2.4. TRAIL and Curcumin Induce Morphological and Molecular Changes Associated with Pyroptosis

**Figure 5:**
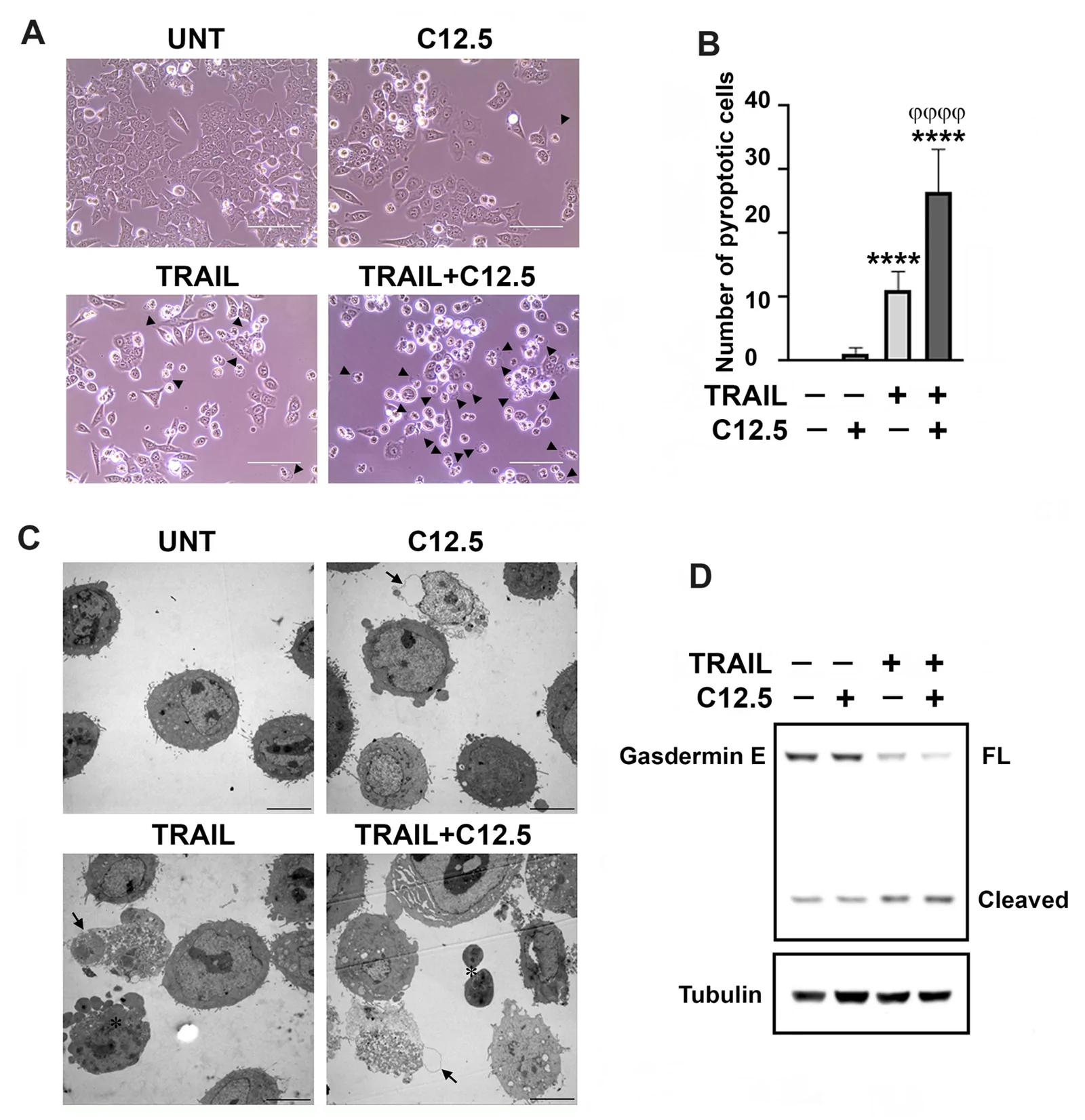
Evaluation of pyroptosis in HCT116 cells treated with TRAIL and curcumin. HCT116 cells were treated for 24 h with TRAIL, alone or in combination with 12.5 µM curcumin, and then analyzed in phase-contrast microscopy (**A**,**B**), in transmission electron microscopy (**C**), and Western Blot (**D**). (**A**) Representative brightfield images of HCT116. Black arrowheads indicate bubbles developing from the cell membrane. Magnification: 40×. The scale bar corresponds to 200 µm. (**B**) HCT116 cells were treated as indicated and cells with membrane bubbles (pyroptotic cells) were scored. Statistical significance vs. untreated: **** *p* < 0.0001. Statistical significance vs. TRAIL: ^φφφφ^ *p* < 0.0001. (**C**) Representative transmission electron microscopy images. Arrows indicate pyroptotic cells. * indicates apoptotic cells. Magnification: 2000×. The scale bar corresponds to 5 µm. (**D**) Representative images of Western Blot results for full-length (FL) and cleaved Gasdermin E expression levels. Tubulin was used as loading control.

HCT116 cells were then observed by phase-contrast microscopy to monitor morphological changes (Figure 5A). Untreated cells showed a normal morphology, while cells with typical pyroptotic morphology were present in cultures treated with TRAIL alone or in combination with curcumin. Of note, the number of pyroptotic cells was significantly higher in HCT116 cells treated with TRAIL in combination with curcumin than TRAIL alone (Figure 5B). The presence of cells exhibiting typical features of pyroptosis, such as formation of large bubbles, disruption of plasma membrane and cell swelling, was also confirmed by transmission electron microscopy (Figure 5C).

Additionally, to confirm morphological observations, we evaluated the cleavage of Gasdermin-E, a molecular hallmark of pyroptosis. As shown in Figure 5D, TRAIL alone or in combination with curcumin was able to induce the cleavage of Gasdermin-E, with a reduction in the full-length form and an increase in the cleaved N-terminus fragment. The reduction was particularly evident in cells treated with TRAIL + 12.5 µM curcumin.

## 3. Discussion

Colorectal cancer (CRC) ranks third in incidence worldwide and second for cancer-related death [23]. Despite the advancements in screening, diagnosis and therapy made over recent decades, incidence and mortality rate are predicted to increase to 3.2 million new cases and 1.6 million deaths by 2040, with a prevalence in countries with high Human Development Index [24]. This correlates with an increasing number of risk factors adding up to genetic predisposition: the aging population in developed countries, sedentary lifestyle, smoking, diet and obesity.

Surgery is considered the main curative therapy if there is the possibility of resecting the tumor with clear margins, but preoperative evaluation of tumor spread is necessary to determine the best therapeutic option. Synchronous metastases are present in ≃20% of newly diagnosed CRCs and up to 55–80% in patients who underwent surgery for metastasis removal. The recommended chemotherapy for CRC is a combination of two or three cytotoxic drugs, which in some cases can be administered in combination with targeted biological agents to hit several critical vital features of the tumor at the same time [25,26]. Despite the efforts, even combined therapies do not guarantee complete cure, generating an urgent need for novel therapeutics to target different mechanisms of tumor growth and metastasis, reduce chemoresistance and minimize relapse.

Several studies have demonstrated that the combination of TRAIL with different compounds [27,28], also of natural origin [29,30], could increase the sensitivity of carcinoma cells to this drug.

In this context, the present study offers an in-depth analysis of the therapeutic activity of curcumin, a natural molecule extracted from turmeric rhizomes, used in combination with TRAIL, whose effects on several cancers have been proved in vitro and in vivo, as well as in several clinical trials [1,31]. Both TRAIL and curcumin have demonstrated a pro-apoptotic action, inducing the extrinsic and intrinsic pathways, respectively [32,33,34,35,36], that converge in the activation of the effector caspases, suggesting that the anti-tumoral activity of the two molecules could be combined to overcome chemoresistance.

In our study we have demonstrated that TRAIL used in combination with curcumin was more effective in inducing apoptosis than TRAIL alone, after both 24 and 48 h of treatment, as previously demonstrated for prostate cancer [31], ovarian cancer [37] and cholangiocarcinoma [38]. Of note, after 48 h, the apoptosis level induced by TRAIL alone showed a significant decrease, probably due to the short half-life of TRAIL which cannot induce a cytotoxic effect for a long period [39,40], while the combinatorial treatment remained at the same level of apoptosis observed after 24 h of treatment. These results suggest that cells sensitized by curcumin continue to undergo apoptosis induced by TRAIL even when the treatment with the cytokine alone decreases its effectiveness.

The increased efficacy of cotreatment to induce apoptosis was also confirmed by protein expression analyses of the apoptotic markers PARP and γ-H2AX (the phosphorylated form of H2AX), since cleavage of PARP and phosphorylation of H2AX are early events in cells undergoing apoptosis.

PARP is an enzyme involved in DNA repair mechanisms, cleaved by active effector caspases during apoptosis [41]. In our results it is evident that PARP cleavage is induced by TRAIL alone or in combination with curcumin at both concentrations, but not by curcumin alone, confirming that curcumin is not sufficient to induce apoptosis in colon cancer cells. Another apoptotic marker analyzed was γ-H2AX, the phosphorylated form at Serine 139 of the histone protein H2AX. It has been demonstrated that this modification at the C-terminus of the protein is involved in apoptosis induced in distinct tumor cells, including colon cancer cells, by pharmacological treatments [42,43]. In our model, H2AX phosphorylation was slightly induced already with 12.5 µM curcumin alone, and significantly with TRAIL alone or in combination with respect to untreated cells. Moreover, in line with the results obtained by apoptosis and cell viability analyses, cotreatment with 12.5 µM curcumin was more effective in inducing H2AX phosphorylation than TRAIL alone. Previous studies have also demonstrated that p53 is involved in γ-H2AX dephosphorylation via induction of phosphatase 1 [42,44], suggesting a pro-survival activity for p53. In our study we observed that the combined treatment lowered p53 phosphorylation induced by treatment with curcumin alone, restoring protein levels to a range comparable to the control. The inhibition of p53 by TRAIL may explain the cotreatment’s ability to induce higher levels of γ-H2AX, and consequently apoptosis, with respect to TRAIL used alone.

TRAIL and curcumin were also able to downmodulate the expression profile of anti-apoptotic proteins belonging to the inhibitors of apoptosis protein (IAP) family, which are usually deregulated in different types of cancers [45], including colon cancer [46], and their levels could predict treatment response and survival rate [47].

XIAP acts as an anti-apoptotic factor directly binding caspases and inhibiting their activity [48], while c-IAP1 and c-IAP2, the two closest paralogs of XIAP, bind caspases but exert anti-apoptotic effects through their E3 ubiquitin ligase activity [49]. Livin and Survivin suppress apoptosis by binding caspases and are highly expressed in human cancers, while they are rarely detected in normal tissue [50,51]. Several studies have demonstrated that these factors exert a critical role in regulating TRAIL resistance in different cancer cells [28,49,52,53], including colon cancer [30,54,55], and that the pro-apoptotic activity of TRAIL could be restored using TRAIL in combination with different molecules.

Consistent with these studies, our results suggest that curcumin is able to sensitize colon carcinoma cells to TRAIL, downmodulating c-IAP1, Livin, Survivin and XIAP, all members of the IAP family. Indeed, cotreatment with curcumin and TRAIL induced a decrease in the expression levels of c-IAP1, Livin and Survivin with respect to untreated cells. Furthermore, the combinatorial treatment downregulated c-IAP1 and Livin even compared to TRAIL used alone, demonstrating a similar effect also for XIAP protein. Moreover, Ge et al. have shown that Livin was able to induce colon cancer progression lowering γ-H2AX levels [56], supporting our results that have shown that cotreatment with TRAIL and curcumin induced an increase in the phosphorylated form of H2AX.

To further investigate the effects of the combination of TRAIL and curcumin, we analyzed the expression of proteins implicated in the apoptotic pathways at different levels.

First, we evaluated the expression profile of BAX, a protein involved in the intrinsic apoptotic pathway [57]. Our results showed that TRAIL alone or in combination with curcumin was able to induce the cleavage of BAX into an 18 kDa fragment (p18 BAX), a potent pro-apoptotic factor capable of inducing cytochrome-c release from mitochondria with a Bcl-2-independent mechanism [58]. In particular, the expression of p18 BAX was significantly elevated in cells treated with TRAIL in combination with curcumin with respect to TRAIL alone, consistent with a potentiating effect of curcumin on TRAIL activity.

We have also evaluated Caspase-3, a pro-apototic effector caspase activated by both the extrinsic and intrinsic apoptotic pathways [59]. As expected, TRAIL alone or in combination with curcumin induced the cleavage of Caspase-3, while curcumin alone was not able to promote its activation [1,60,61,62]. These results also support the induction of PARP cleavage by treatments, given that it has been demonstrated that Caspase-3 is implicated in this apoptotic mechanism [63].

Recent studies have also shown that Caspase-3, typically recruited in the process of apoptosis, is involved in an alternative signaling pathway leading to pyroptosis in cancer cells [64,65]. Pyroptosis is a programmed cell death mediated by proteins of the Gasdermin (GSDM) family. Gasdermins form pores on the cellular membrane, causing cell swelling and eventually rupture of the cellular membrane, with the release of intracellular content into the extracellular space, triggering an inflammatory response, with the recruitment and the activation of immune cells at the site [66]. Pyroptosis can be induced by the BAK/BAX/Caspase-3/GSDM-E axis [61], in which Caspase-3 mediates GSDM-E cleavage, releasing its N-terminal fragment that aggregates with other GSDM-E N-terminal fragments to form pores on the membrane. GSDM-E also acts in different tumors, including colon carcinoma, activating immune response cells [67].

Our results confirm the induction of pyroptosis as suggested by the morphological changes observable with a light microscope and by electron microscopy. Pyroptotic cells can be identified due to the presence of a bubble-like protrusion of the cytoplasm, characteristic of swollen pyroptotic cells [68,69]. TRAIL alone was able to induce pyroptosis, but the effect was enhanced when TRAIL was concurrently used with curcumin. Indeed, the number of pyroptotic cells was significantly higher in samples treated with TRAIL and curcumin with respect to TRAIL used alone, showing an increased effect of the combinatorial treatment. To further confirm these observations, we studied pyroptosis from a molecular perspective, analyzing the expression of GSDM-E. As mentioned above, GSDM-E is downstream of the effector Caspase-3, activated by both the extrinsic and intrinsic apoptotic pathways, and thus it represents an ideal marker to determine the pyroptotic activity of TRAIL and curcumin. In particular, by Western Blot analysis, we determined the presence of the cleaved N-terminal fragment of GSDM-E, released after cleavage by Caspase-3 and forms the pyroptotic pores on the cellular membrane.

Based on our data, it can be hypothesized that the induction of the potent pro-apoptotic factor p18 BAX, and the consequent release of cytochrome-c, could trigger a positive feedback loop able to increase the activation of Caspase-3, and to induce a Caspase-3-mediated pyroptotic response in colon cancer cells, as previously demonstrated in colon cancer cells treated with lobaplatin [68]. Furthermore, our results suggest that sufficient activation of TRAIL signaling by curcumin could lead to both apoptosis and pyroptosis, depending on the state of each cell, suggesting that induction of apoptosis should not be the sole goal of anti-tumor chemotherapy and that this could help to circumvent resistance to apoptosis and promote anti-tumoral immune response.

Since many tumors develop resistance to classical cell death-inducing therapies, studying molecules or therapeutic combinations able to activate different pathways of cell death can represent an opportunity to potentiate current therapies and avoid tumor multidrug resistance. In this context, curcumin can potentiate the action of TRAIL to counteract cell growth and proliferation and overcome resistance to TRAIL. Curcumin alone does not show a strong pro-apoptotic effect on CRC cells, however its ability to target different key pathways of tumor cells plays a central role in combined treatment with TRAIL.

This study has some limitations. First, it is an in vitro proof of concept in a colorectal cancer cell line. Second, the curcumin concentrations tested (8 and 12.5 µM), at which curcumin alone induced little or no apoptosis, cannot be directly extrapolated to plasma or tissue levels achievable in vivo, given the poor bioavailability of curcumin [16]. Third, the stability and bioavailability of both curcumin and TRAIL, which has a short half-life [39,40], were not assessed. Nevertheless, the finding that curcumin sensitizes cells to TRAIL at low micromolar concentrations supports the rationale that combination strategies may allow lower curcumin doses to be used.

To address these limitations, we plan to deliver curcumin through nanoparticle-based formulations to improve its bioavailability and enhance its combination with TRAIL [70,71]. The nanoparticle-encapsulated curcumin will be tested in combination with TRAIL in intestinal organoid models, which better recapitulate the complexity of the intestinal epithelium than conventional 2D cultures. Subsequent in vivo studies will be needed to assess the pharmacokinetics of both compounds.

Mechanistic follow-up will include Caspase-3 inhibition and GSDM-E silencing to test the Caspase-3/GSDM-E axis, together with analysis of DR4/DR5, c-FLIP, caspase-8/-9, cytochrome-c release and LDH release.

## 4. Materials and Methods

### 4.1. Cell Culture and Treatments

Curcumin (*Curcuma longa* L.) was purchased from Merck (Darmstadt, Germany) and was dissolved in DMSO according to the producer’s instructions. Recombinant TRAIL was prepared as described below. Briefly, recombinant human histidine 6-tagged TRAIL_(114–281)_ was produced in bacteria BL21, transformed with a pTrc-His6 TRAIL vector. The gene sequence (residues 114 to 281), cloned in frame to a histidine 6-tag, was preceded by an inducible promoter, that was stimulated when bacteria were in logarithmic-phase growth.

The subsequent extraction of the protein occurred through sonication and different buffers, while purification was obtained by affinity chromatography on Ni^2+^ affinity resin. Purification consisted of three steps: binding of the protein to resin by histidine 6-tag, washing of the unbound, and elution of the purified protein. Finally, the preparations were dialyzed for 48 h in saline solution, aliquoted and stored at −80 °C. To quantify the presence of bacterial endotoxin, TRAIL preparations were tested with the Chromogenic Limulus Amebocyte Lysate (LAL) Test (LONZA, Basel, Switzerland) and results were always lower than 0.1 EU/mL. The purity of each TRAIL preparation was evaluated by loading 2 μg recombinant TRAIL in 15% sodium dodecyl sulfate–polyacrylamide gel electrophoresis (SDS-PAGE), containing (reducing condition) or not containing (non-reducing condition) 5% β-mercaptoethanol. The gels were then stained with the gelcode Silver SNAP Staining kit (ThermoFisher Scientific Inc., Waltham, MA, USA). Finally, the biologic activity of TRAIL preparations was tested on the TRAIL-sensitive HL-60 cell line by propidium iodide (PI) staining and flow cytometric analysis of apoptosis.

The colorectal cancer cell line HCT116, purchased from ATCC (CCL-247EMT™, ATCC^®^, Manassas, VA, USA), was cultured in DMEM High Glucose (CARLO ERBA Reagents SAS, Val de Reuil, France) supplemented with 10% fetal bovine serum (FBS, Merck) and 1% penicillin–streptomycin (Merck). Cells were maintained in a humidified incubator at 37 °C with 5% CO_2_. Then, 24 h after seeding, HCT116 cells were treated with TRAIL 500 ng/mL and curcumin, 8 µM and 12.5 µM, or their combinations for 24 and 48 h.

Cells were then collected for viability assessment, apoptosis analysis, Western Blotting assays and ultrastructural analysis by electron microscopy.

### 4.2. Cell Viability Assay

HCT116 cells were plated in 96-well plates at a density of 4500 cells/well, and their viability was assessed at 24 and 48 h after treatment using the colorimetric MTT assay (Roche, Basel, Switzerland) according to the manufacturer’s protocol. The absorbance was read at 570 nm and at 650 nm as a reference wavelength for background subtraction using a TECAN Infinite^®^ M Plex microplate reader (Tecan Trading AG, Männedorf, Switzerland).

### 4.3. Apoptosis Evaluation by Flow Cytometry

For apoptosis evaluation, HCT116 cells were seeded in 6-well plates at a density of 200,000 cells/well. After 24 and 48 h of treatment, cells were collected by trypsinization, washed twice with a phosphate-based buffer (PBS 1X), centrifuged at 1200 rpm for 5 min, and prepared for flow cytometry analysis. To evaluate the cytotoxicity of the treatments, cells were stained with annexin V-FITC and PI (Beckman Coulter Inc., Brea, CA, USA) according to the manufacturer’s protocol. The acquisition was performed using a FACSCalibur™ Flow Cytometer (BD Biosciences, San Josè, CA, USA) and the data were analyzed with the Flow Jo Software, version 9.9.6 (Tree Star, Ashland, OR, USA).

### 4.4. Proteome Profiler Human Apoptosis Array

Apoptosis-related protein expression was assessed using the Proteome Profiler Human Apoptosis Array Kit (R&D Systems, Minneapolis, MN, USA) following the manufacturer’s instructions. HCT116 cells were lysed and 400 µg of total protein from each sample was incubated with the array membranes overnight at 4 °C on a rocking platform. After washing, membranes were incubated with the biotinylated detection antibody cocktail, followed by streptavidin–HRP. Chemiluminescent signals were developed using the provided Chemi Reagent Mix and acquired using iBrightTM FL 1500 Imaging System instrument (ThermoFisher Scientific Inc.). The quantitative analysis of the spots’ intensity was carried out with the iBright Analysis Software, Desktop Version 5.2.2 (ThermoFisher Scientific Inc.) associated with the instrument and the resulting values were normalized to the internal reference spots present on each membrane.

### 4.5. Western Blotting

The analysis of protein expression was performed by seeding the HCT116 cells in a T-25 flask at a density of 2 × 10^6^ cells/flask. Following 24 h of treatments, cells were collected, washed with PBS 1X and centrifuged at 1200 rpm for 5 min. The pellets for each condition were frozen at −80 °C and then lysed in RIPA buffer (50 mM Tris pH 7.5, 150 mM NaCl, 0.1% SDS, 1% Nonidet P-40, 0.25% sodium deoxycholate), supplemented with a cocktail of phosphatase and protease inhibitors (ThermoFisher Scientific Inc.). The quantification of total proteins for each lysate was performed with the Pierce BCA Assay Kit (ThermoFisher Scientific Inc.). Then, 50 µg of total proteins were subjected to SDS-PAGE followed by transfer to a nitrocellulose membrane (Merck KGaA).

The membranes were incubated with primary antibodies targeting Bcl-2, PARP (both from Santa Cruz Biotechnology; Dallas, TX, USA), γ-H2AX (Serine139), Caspase-3, BAX, Gasdermin E (all from Cell Signaling Technology, Danvers, MA, USA) and β-tubulin (Merck). After washing two times with TBST 0.1%, the membranes were incubated with secondary anti-mouse or anti-rabbit antibodies. After the incubation period, the protein bands were detected with chemiluminescence using SuperSignal^TM^ West Pico PLUS Chemiluminescent Substrate kit (ThermoFisher Scientific Inc.). Finally, the images were acquired with the iBright^TM^ FL 1500 Imaging System instrument (ThermoFisher Scientific Inc.), while the analysis was carried out with the iBright Analysis Software, Desktop Version 5.2.2 (ThermoFisher Scientific Inc.) associated with the instrument.

### 4.6. Transmission Electron Microscopy (TEM) Analysis

For electron microscopy analysis, HCT116 cells were seeded in a T-25 flask at a density of 2 × 10^6^ cells/flask. After 24 h of treatment, cells were collected by trypsinization, washed twice with a phosphate-based buffer, and centrifuged at 1200 rpm for 5 min. Then, the pellets were fixed for 3 h at room temperature in 2.5% glutaraldehyde in 0.1 M phosphate-buffered saline (PBS1X, pH 7.4). Subsequently, two washes were performed in PBS1X and post-fixed for 1 h at room temperature in 2% buffered osmium tetroxide. After these steps, the samples were dehydrated with increasing concentrations of acetone and included in Araldite epoxy resin (Durcupan ACM, Fluka, Sigma Aldrich Co., St. Louis, MO, USA), according to the standard protocols. Subsequently, semi-thin sections (1.5 μm) were obtained and cut with an ultramicrotome (Reichert Ultracut S, Leica, Wetzlar, Germany), using glass blades. The semi-thin sections were then stained with a 1% toluidine blue solution and observed under an optical microscope (Nikon Eclipse E800, Amstelveen, The Netherlands) to identify areas of interest. Once these areas had been identified, ultrathin sections (90 nm) were obtained using an ultramicrotome (Reichert Ultracut S) and counter-stained with uranyl acetate in a saturated solution and lead citrate according to Reynolds. After sample preparation, observations were carried out with a transmission electron microscope, ThermoFisher Talos L120C G2 (S)TEM (ThermoFisher Scientific Inc.).

### 4.7. Statistical Analysis

Data are expressed as mean ± standard deviation (SD) of at least three independent experiments. Statistical analysis was performed using GraphPad Prism software, Version 8.0.1 (GraphPad Software, Boston, MA, USA). Statistical significance was determined by one-way ANOVA followed by Tukey correction for multiple comparisons. A *p* value < 0.05 was considered statistically significant.

## 5. Conclusions

In conclusion, this study shows that curcumin, particularly at 12.5 µM, markedly enhances the pro-apoptotic activity of TRAIL in HCT116 colorectal cancer cells, sustaining apoptosis levels even after 48 h, when TRAIL alone loses efficacy. Mechanistically, the combined treatment increased PARP and Caspase-3 cleavage, γ-H2AX phosphorylation and BAX activation, while downregulating the anti-apoptotic IAP family members c-IAP1, Livin, Survivin and XIAP. Notably, TRAIL and curcumin cotreatment also induced pyroptosis, as shown by characteristic morphological changes and increased Gasdermin-E cleavage, suggesting that cell death may involve both apoptotic and pyroptotic pathways. Taken together, these findings support curcumin as a promising sensitizing agent to help address TRAIL resistance in this experimental model of colorectal cancer, and warrant further in vivo studies to validate this combinatorial strategy and to address the bioavailability limitations of both compounds ahead of clinical translation.

Taken together, these findings suggest that curcumin may act as a sensitizing agent for TRAIL in this experimental model of colorectal cancer. Given the known limitations of curcumin (PAINS behavior, poor bioavailability, lack of conclusive clinical efficacy), further studies, including nanoparticle-based delivery tested in organoid models to confirm specificity, and subsequent in vivo validation, are needed before any clinical translation.

## Data Availability

The data presented in this study are available in the article and in the Supplementary Materials. Further data are available on request from the corresponding author.

## Supplementary Materials

The following supporting information can be downloaded at https://www.mdpi.com/article/10.3390/ijms27219444/s1.

## Abbreviations

The following abbreviations are used in this manuscript:

CRC	Colorectal cancer
TRAIL	Tumor necrosis factor-related apoptosis-inducing ligand
DR4/DR5	Death receptor 4/5
DcR1/DcR2	Decoy receptor 1/2
c-FLIP	Cellular FLICE-like inhibitory protein
IAP	Inhibitor of apoptosis protein
c-IAP1/c-IAP2	Cellular inhibitor of apoptosis protein 1/2
XIAP	X-linked inhibitor of apoptosis protein
PARP	Poly(ADP-ribose) polymerase
γ-H2AX	Phosphorylated histone H2AX (Ser139)
BAX	BCL2-associated X protein
BAK	BCL2 antagonist/killer
GSDM-E	Gasdermin E
NF-κB	Nuclear factor kappa-light-chain-enhancer of activated B cells
PI3K/Akt	Phosphoinositide 3-kinase/protein kinase B
STAT3	Signal transducer and activator of transcription 3
HDI	Human Development Index
FBS	Fetal bovine serum
DMEM	Dulbecco’s Modified Eagle Medium
ATCC	American Type Culture Collection
DMSO	Dimethyl sulfoxide
PBS	Phosphate-buffered saline
FITC	Fluorescein isothiocyanate
PI	Propidium iodide
HRP	Horseradish peroxidase
RIPA	Radioimmunoprecipitation assay (buffer)
