# Supplementary material for: Curcumin Sensitizes Colorectal Cancer Cells to TRAIL-Mediated Apoptosis
Source: Int J Mol Sci. 2026 Oct 23;27(21):9444. doi: 10.3390/ijms27219444 (PMC13763032; doi:10.3390/ijms27219444)
Supplement: Supplementary file 1 [file ijms-27-09444-s001.zip › ijms-4612627-supplementary.pdf]

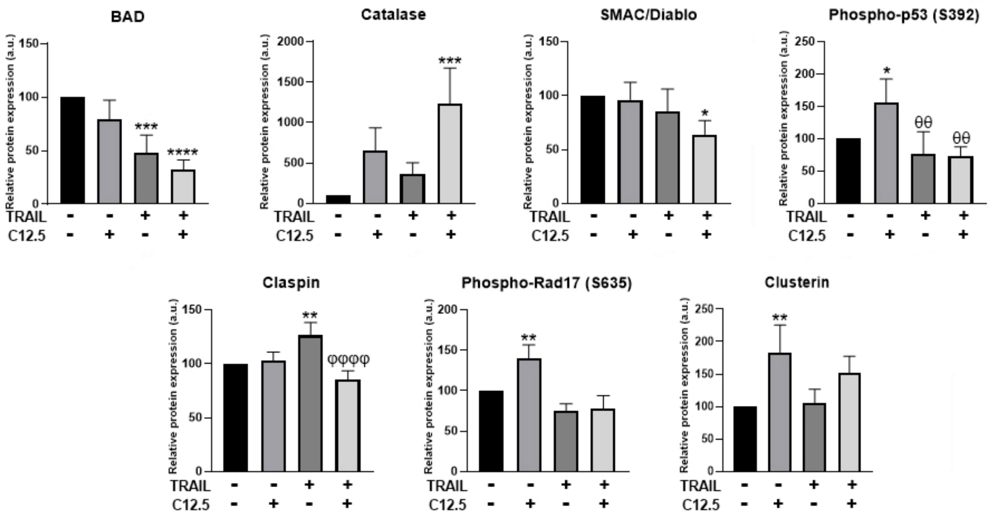

Figure S1. Evaluation of multiple apoptosis-related proteins in HCT116 after 24 hours of treatment. HCT116 cells were treated for 24 hours with TRAIL alone or in combination with curcumin 12.5µM. Results of at least three independent experiments are presented as % respect to untreated set to 100% and are reported as mean±SD. Statistical significance vs Untreated: * $p < 0.05$; ** $p < 0.01$; ***; $p < 0.001$; ****; $p < 0.0001$. Statistical significance vs TRAIL: $^{\varphi\varphi\varphi\varphi}$ $p < 0.0001$. Statistical significance vs C12.5: $^{\theta\theta}$ $p < 0.01$.
